# Nrf2 functions as a pyroptosis-related mediator in traumatic brain injury and is correlated with cytokines and disease severity: a bioinformatics analysis and retrospective clinical study

**DOI:** 10.3389/fneur.2024.1341342

**Published:** 2024-02-09

**Authors:** Gengshui Zhao, Jianfei Zhao, Jiadong Lang, Guozhu Sun

**Affiliations:** ^1^Department of Neurosurgery, The Second Hospital of Hebei Medical University, Shijiazhuang, China; ^2^Department of Neurosurgery, Harrison International Peace Hospital Affiliated to Hebei Medical University, Hengshui, China; ^3^Department of Neurosurgery, The People's Hospital of Shijiazhuang City, Shijiazhuang, China

**Keywords:** bioinformatics, Nrf2, traumatic brain injury, cytokines, pyroptosis

## Abstract

**Background:**

Traumatic brain injury (TBI) is a serious hazard to human health. Evidence has accumulated that pyroptosis plays an important role in brain trauma. The aim of this study is to screen potential key molecules between TBI and pyroptosis, and further explore their relationships with disease severity and cytokines.

**Methods:**

To acquire differentially expressed genes (DEGs) before and after brain injury, the GSE89866 dataset was downloaded from the Gene Expression Omnibus (GEO) database. Meanwhile, pyroptosis-related genes were obtained from the GeneCards database, and the intersected genes were identified as differentially expressed pyroptosis-related genes (DEPGs). Moreover, the hub genes were screened via four algorithms (namely Maximum Clique Centrality, Edge Percolated Component, BottleNeck and EcCentricity) in Cytoscape software. Blood levels of Nrf2 were measured by ELISA using a commercially available kit. Finally, we further investigated the correlation between Nrf2 levels and medical indicators in TBI such as clinical characteristics, inflammatory cytokines, and severity.

**Results:**

Altogether, we found 1,795 DEGs in GSE89866 and 98 pyroptosis-related genes in the GeneCards database. Subsequently, four hub genes were obtained, and NFE2L2 was adopted for further clinical study. By using Kruskal-Wallis test and Spearman correlation test, we found that the serum Nrf2 levels in severe TBI patients were negatively correlated with GCS scores. On the contrary, there was a positive correlation between serum Nrf2 levels and pupil parameters, Helsinki CT scores, IL-1 β and IL-18.

**Conclusions:**

In summary, bioinformatic analyses showed NFE2L2 plays a significant role in the pathology of TBI. The clinical research indicated the increase in serum Nrf2 levels was closely related to the severity of trauma and cytokines. We speculate that serum Nrf2 may serve as a promising biochemical marker for the assessment of TBI in clinical practice.

## 1 Introduction

Traumatic brain injury (TBI), especially severe traumatic brain injury (sTBI), is a major public health problem that could result in death or disability. Annually, over 80,000 persons die from TBI in Europe, and in the United States TBI lead to 53,000 deaths (1–3). Pyroptosis, as a form of programmed cell death, also plays an important role in the development and prognosis of many neurological diseases, including TBI (4). Currently, some studies has noticed the pathophysiological changes related to pyroptosis after TBI, especially the changes of pyroptosis-related cytokines (such as IL-1β and IL-18) (5). However, the exact molecular basis between TBI and pyroptosis is not entirely clear.

In recent years, bioinformatic approaches have been widely used in neuroscience research. More and more studies have focused on the roles of serum markers in the treatment of TBI. Our study analyzed potential pyroptosis-related genes in TBI, which laid the foundation for subsequent clinical study. The Glasgow Coma Scale (GCS), pupil parameters and Helsinki CT score are often used to describe brain injury and evaluate its prognosis (6, 7). The pathophysiological process secondary to trauma is important and complex, and many molecules are involved to influence the progression and outcome of the disease. There are numerous danger/damage-associated molecular patterns (DAMPs) released after trauma that trigger inflammasome assembly (8). *Schaefer* indicated that inflammasome activation could result from extracellular DAMPs released by surrounding stressed or dying cells, or via intracellular nucleic and mitochondrial DAMPs translocating into the cytosol in response to cell stress (9). Using a concentration gradient of DAMPs and cytokines, inflammation mediated by inflammasome recruits and activates immune cells (10). Hegdekar et al. (11) revealed that defects in microglial autophagy following injury were associated with decreased phagocytic clearance of DAMPs responsible for activation of the cellular innate immune responses.

In our study, we obtained the genes with different expressions through the public database, and then screened the crosstalk genes by intersecting with the pyroptosis-related genes. The second half of our article investigated the correlation between serum levels of critical biomolecule and medical indicators in TBI such as clinical characteristics, inflammatory cytokines, and severity.

## 2 Methods

### 2.1 Datasets and study design

**Figure 1:**
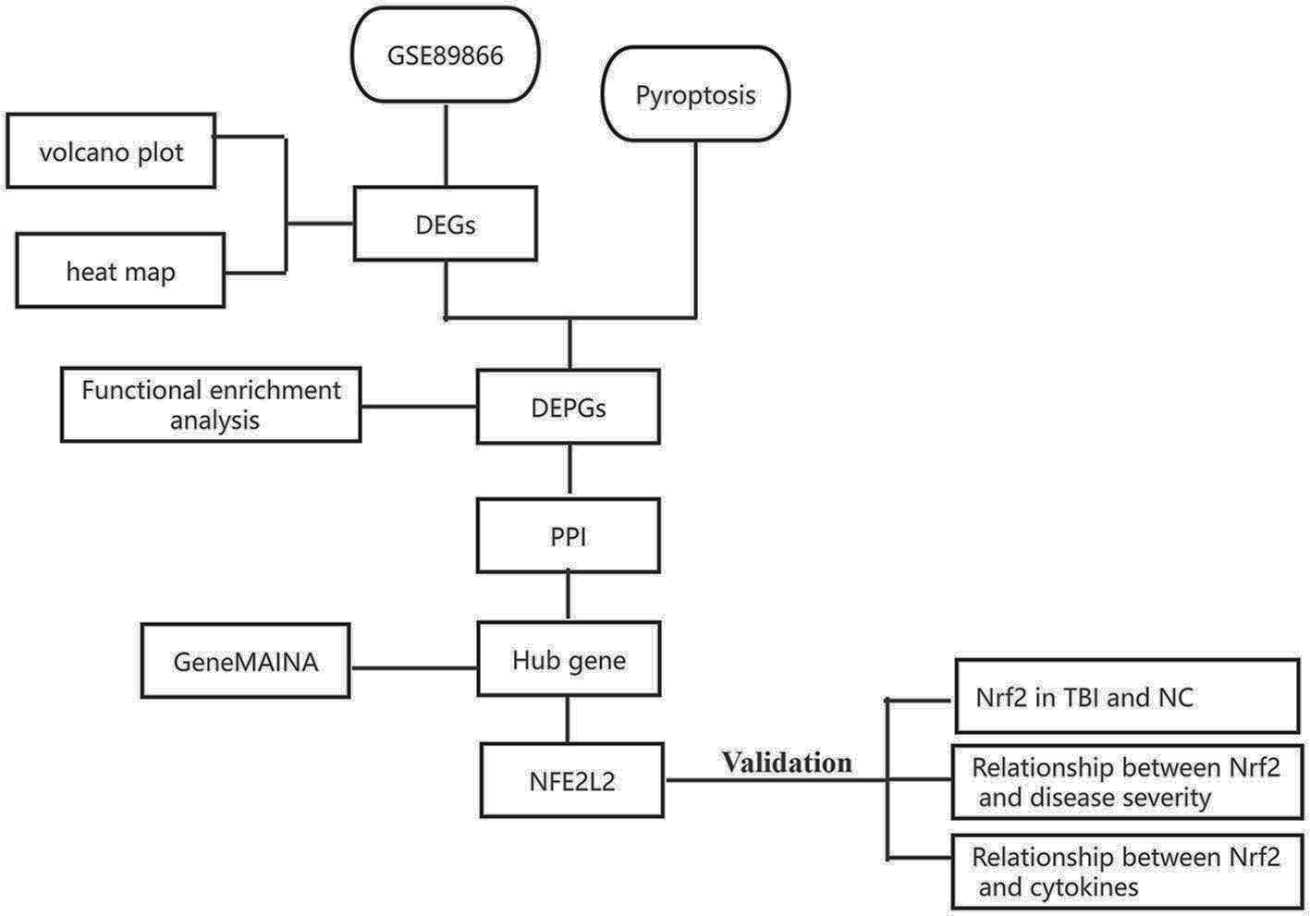
The workflow of our study. DEGs, differentially expressed genes; DEPGs, differentially expressed pyroptosis-related genes; PPI, protein–protein interaction; NC, normal control.

The TBI gene expression microarray dataset was downloaded from the National Center for Biotechnology Information (NCBI) Gene Expression Omnibus (GEO, www.ncbi.nlm.nih.gov/geo/) database with the accession number GSE89866. This dataset, which was based on the GPL16791 Illumina HiSeq 2500 platform (Homo sapiens), contained TBI samples and no-TBI controls. Gene expression data were derived from the participants' serum. Blood samples were taken daily for three consecutive days after injury, and the sequencing results were compared with each participant's own baseline. A diagram of the analytical workflow was described in Figure 1.

### 2.2 Identification of differentially expressed pyroptosis-related genes

NetworkAnalyst (https://www.networkanalyst.ca/) is an R-based analysis tool available online. We utilized NetworkAnalyst to compare gene expression profiles between different groups in GSE89866. Genes with an adjusted *p*-value < 0.05 were considered differentially expressed (DEGs). The volcano plot and heat map were created by an online tool (www.bioinformatics.com.cn).

Meanwhile, pyroptosis-related genes were extracted from GeneCards (Version 5.18.0, https://www.Genecards.org/). The search strings for pyroptosis in the GeneCard were listed in Supplementary Appendix 1. The differentially expressed pyroptosis-related genes (DEPGs) were obtained by overlapping DEGs and the pyroptosis-related genes. A Venn diagram was used to visualize the DEPGs (http://bioinformatics.psb.ugent.be/webtools/Venn/).

### 2.3 Functional enrichment analysis

To explore the biological significance of the DEPGs, Gene Ontology (GO) annotation and Kyoto Encyclopedia of Genes and Genomes (KEGG) pathway enrichment analyses were performed by DAVID tools (https://david.ncifcrf.gov/). The entries with *p*-values < 0.05 were considered significant.

### 2.4 Protein–protein interaction network construction, hub genes identification and GeneMAINA analysis

The proteins encoded by the DEPGs were used to construct the protein–protein interaction (PPI) network by Search Tool for the Retrieval of Interacting Genes (STRING, http://string-db.org). Cytoscape 3.6.1 (https://cytoscape.org) was utilized to visualize the results. Moreover, we adopted CytoHubba, a Cytoscape plug-in, to screen hub genes. Based on the results of four widely used algorithms—Maximum Clique Centrality (MCC), Edge Percolated Component (EPC), BottleNeck, and EcCentricity—the overlapped genes were identified as hub genes (3, 4). Visualization of the hub gene network was generated via GeneMAINA (http://genemania.org).

### 2.5 Clinical study population

This study was conducted in accordance with the Helsinki Declaration (2013 revision) and was reviewed and approved by the Ethics Committee of Harrison International Peace Hospital. To protect patient privacy, all personal information was removed, and the review committee has waived the requirement of informed consent. The reporting of this study conforms to STROBE guidelines.

Patients diagnosed with sTBI (GCS score ≤ 8) were included in this study with the following inclusion criteria: ① a clear history of head trauma; ② age ≧ 18 years old; ③ blunt trauma; ④ hospital admission within 24 h after trauma; ⑤ Injury Severity Score (ISS) < 9 for injuries to other parts except the brain; ⑥ the CT image data after injury were complete. The exclusion criteria were as follows: ① previous neurological diseases such as stroke, Parkinson's disease, intracranial tumors, etc.; ② other specific diseases, such as severe infections within the last month; autoimmune diseases; hemorrhagic diseases or bleeding tendency; severe liver, kidney or heart disease; and malignant tumors; ③ pregnancy; ④ severe intellectual disability, mental illness, or other diseases that affect the patient's mental state.

The healthy controls, matched for sex and age, had no chronic diseases such as hypertension, diabetes, coronary heart disease, and no history of acute infection or medication in the month prior to testing. Routine blood tests, liver, and kidney function tests showed no obvious abnormalities.

### 2.6 Collection of clinical and radiological information

Data related to patient baseline demographic and clinical characteristics extracted from medical records, including age, sex, smoking history, alcohol consumption history, past medical history, cause of injury, pupil parameters, and body mass index (BMI), etc. Pupil evaluation was referred to the previous literature (Supplementary Table 1) (6, 12, 13). Pupil reactivity was coded into three subgroups: (1) brisk (both pupils constricted identically with light shone into either eye alone); (2) sluggish (delayed pupil constriction in reaction to light); (3) fixed (no response to a light stimulus, regardless of which eye was being stimulated). Similarly, pupil size was coded into three subgroups: (1) normal (bilateral equal non-dilated pupil size); (2) anisocoric (unequal pupil sizes); (3) bilateral dilated.

At the same time, the Helsinki CT score was calculated from head CT scan images to assess radiological severity of head trauma (7). Positive radiological findings included epidural hematoma, subdural hematoma, intracerebral hematoma, intraventricular hemorrhage, lesion volume, and morphology of suprasellar cisterna. The scores range from −3 to 14, with higher scores indicating greater severity. All CT measurements were based on the Picture Archive Communication System (PACS). The specific scores were determined by the researcher who was not familiar with clinical data using thin slice CT scans of the head within 24 h after injury.

### 2.7 Determination of serum nuclear factor red blood cell 2 related factor 2 and cytokines

The candidate gene NFE2L2 was screened by aforementioned bioinformatics methods. To explore the relationship between NFE2L2 and cytokines, the expression levels of nuclear factor red blood cell 2 related factor 2 (Nrf2), IL1β and IL18 were detected by enzyme-linked immunosorbent assay (ELISA). Blood samples were obtained from the median cubital vein (both patients and healthy controls). All samples for Nrf2 testing were obtained at 24 h after injury. After centrifugation at 3,000 rpm for 10 min, serum samples were immediately subjected to the following analysis, or immediately stored at −80°C for further analysis.

According to the instructions of the ELISA kit (Elabscience; Wuhan, China), the same technician without knowledge of clinical and radiological information measured the serum levels of Nrf2, IL-1 β, and IL-18. In short, samples and standards were incubated in a pre-coated 96-well plate for 90 min, followed by a biotinylated antibody working solution for 1 h. After incubation, all holes were washed and incubated with antibodies conjugated with horseradish peroxidase for 30 min, followed by a substrate solution in the dark for 30 min. Finally, the termination solution was added to stop the reaction and the plate reading was immediately taken at 450 nm on the microplate reader (Thermo Fisher Scientific, USA).

### 2.8 Statistical analysis

The significance of differences in Nrf2 protein levels between sTBI and healthy controls was examined using the *t*-test. One-way analysis of variance or Kruskal–Wallis test was used for comparisons as appropriate. Spearman correlation test was used to investigate the correlation between serum Nrf2 protein and cytokines (IL-1β, IL-18). Analysis was performed using IBM SPSS Software version 25.0 (IBM Corp., Armonk, NY, USA) and GraphPad Prism 7 (GraphPad Software, San Diego, CA, USA). Results at *p* < 0.05 were considered statistically significant.

## 3 Results

### 3.1 Identification of DEPGs

**Figure 2:**
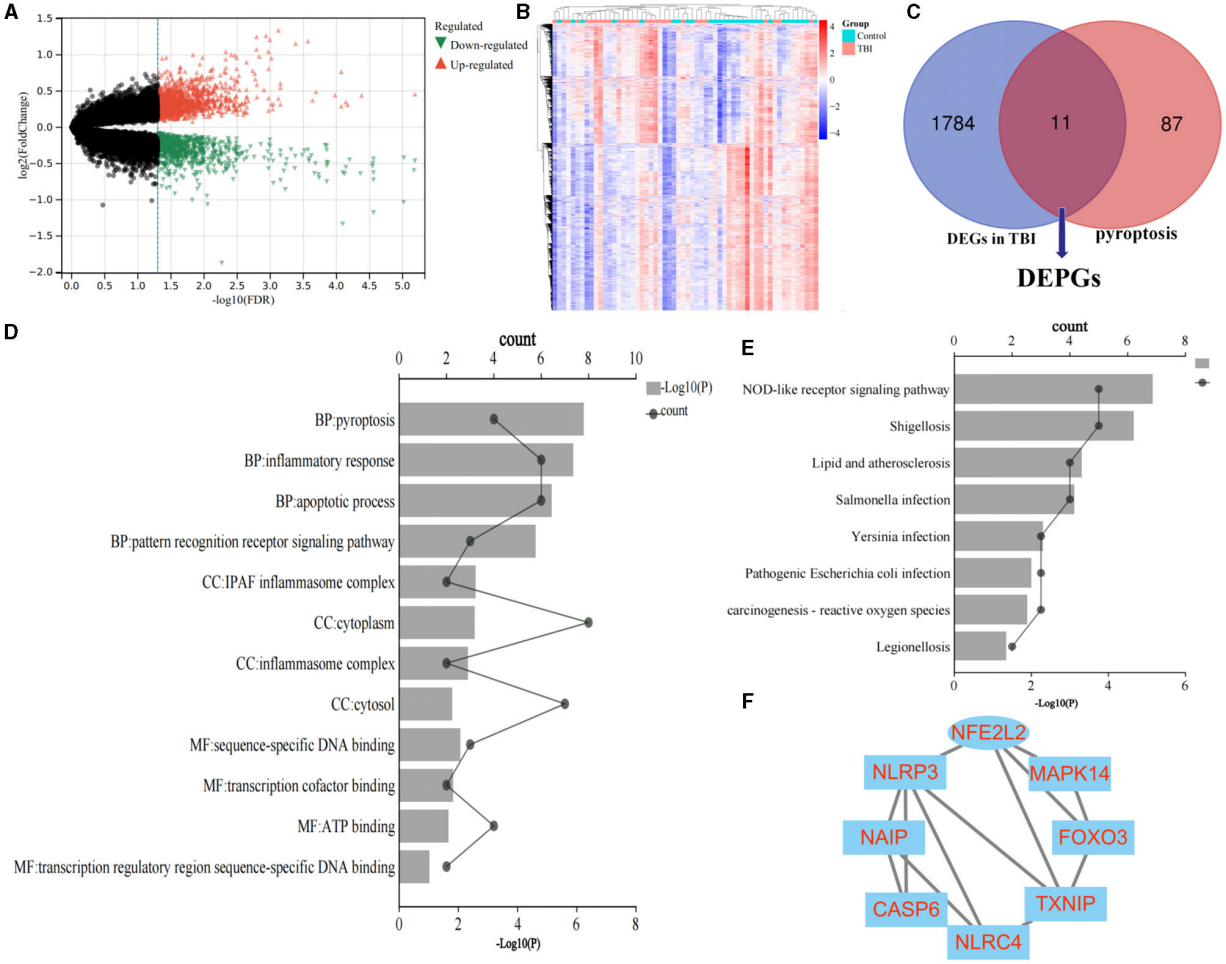
Identification of DEPGs and screening of hub genes. **(A)** Volcano plot shows the DEGs. **(B)** The heat map. **(C)** Identification of DEPGs. **(D)** GO terms of DEPGs. **(E)** KEGG terms of DEPGs. **(F)** PPI network construction. DEGs, differentially expressed genes; DEPGs, differentially expressed pyroptosis-related genes; GO, Gene Ontology annotation; KEGG, Kyoto Encyclopedia of Genes and Genomes.

Based on the aforementioned screening criteria, 1,795 DEGs were obtained in GSE89866. The volcano plot and heat map are displayed in Figures 2A, B. Meanwhile, 98 pyroptosis-related genes were obtained from the GeneCards database (relevance score ≥2, Supplementary Table 2). The intersection of the above two gene sets was selected after Venn diagram analysis, and 11 overlapped genes were defined as DEPGs. The Venn diagram is shown in Figure 2C, and the detailed information on DEPGs is listed within Supplementary Table 3.

### 3.2 Functional enrichment analysis of DEPGs

GO terms include biological processes (BP), cellular components (CC), and molecular functions (MF). As shown in Figure 2D, the top four significant GO terms were chosen according to *p*-value. In terms of GO analysis, DEPGs were mainly enriched in pyroptosis (*p* = 0.00000058), inflammatory response (*p* = 0.0000013) and sequence-specific DNA binding (*p* = 0.0086).

Likewise, as indicated by the results presented in Figure 2E, the significant enriched KEGG terms contained NOD-like receptor signaling pathway (*p* = 0.0000071) and Chemical carcinogenesis—reactive oxygen species (*p* = 0.013). These results demonstrated that chemokines and cytokines may play essential roles in the pathology of TBI. All of the GO terms and KEGG pathways were included in Supplementary Table 4.

### 3.3 PPI network construction, hub gene identification and GeneMAINA analysis

**Figure 3:**
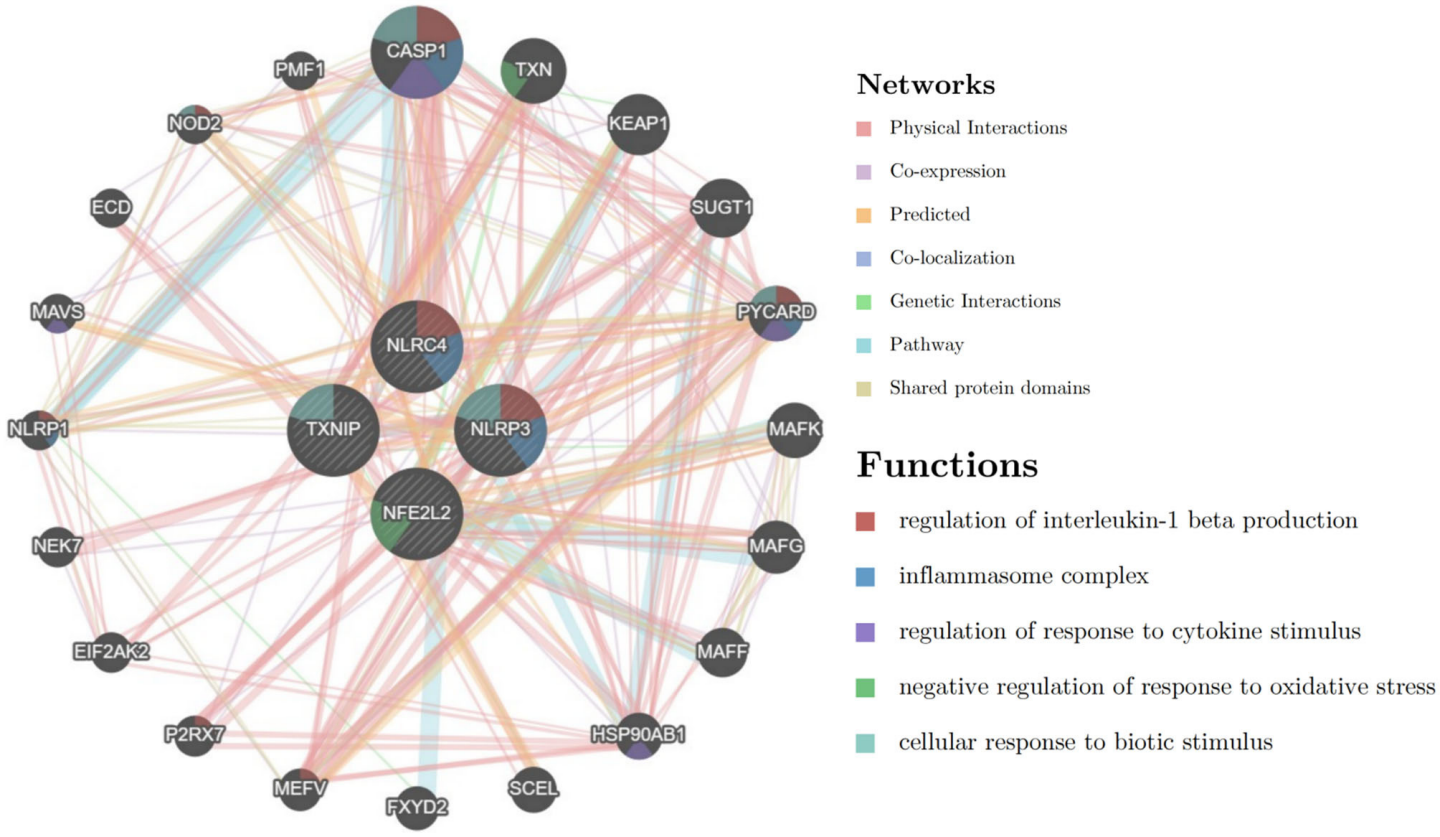
The functional interaction networks using the GeneMANIA database.

The PPI network of DEPGs was analyzed according to the STRING database, including eight nodes and 13 edges (minimum required interaction score: 0.4, Figure 2F). To screen important nodes in the network, all nodes were ranked by the aforementioned methods in CytoHubba. Notably, the top four genes were consistent across methods, namely NFE2L2, TXNIP, NLRP3, and NLRC4 (Supplementary Table 5). The detailed description of the top four genes was provided in Table 1. To further explore these genes, we performed the functional interaction networks using the GeneMANIA database. Functional analysis revealed that these genes were involved in regulation of interleukin-1 beta production, inflammasome complex and regulation of response to cytokine stimulus (Figure 3). The results of functional analysis were consistent with previous studies (14, 15). Based on gene background screening and Nrf2 expression values recorded in medical records, we identified NFE2L2 as the candidate gene for further clinical study.

**Table 1:** The details of the hub genes.

**No**.	**Gene symbol**	**Description**	**Category**	**Function**
1	NFE2L2	NFE2 like BZIP transcription factor 2	Protein coding	Binds to antioxidant response (ARE) elements present in the promoter region of many cytoprotective genes
2	TXNIP	Thioredoxin interacting protein	Protein coding	Acts as an oxidative stress mediator by inhibiting thioredoxin activity or by limiting its bioavailability
3	NLRP3	NLR family pyrin domain containing 3	Protein coding	Mediates inflammasome activation in response to defects in membrane integrity, leading to secretion of inflammatory cytokines
4	NLRC4	NLR family CARD domain containing 4	Protein coding	Key component of inflammasomes that indirectly senses specific proteins from pathogenic bacteria and fungi.

### 3.4 Characteristics of the study population

Table 2 summarized the demographic and clinical data of 81 patients with sTBI and 40 normal controls (NC) included in this study. The sTBI group included 46 males (56.8%) and 35 females (43.2%) with an age range of 18–89 years (mean ± standard difference, 52.48 ± 16.68 years), while the NC group consisted of 21 males (52.5%) and 19 females (47.5%) with an age range of 26–76 years (mean ± standard deviation, 52.18 ± 13.10 years). There were no statistically significant differences in age, sex, BMI, tobacco and alcohol exposure, and underlying diseases between sTBI patients and NC group.

**Table 2:** Baseline demographic and clinical characteristics in 81 patients with sTBI and 40 normal controls.

**Characteristic**	**sTBI (*n* =81)**	**NC (*n* = 40)**	**Statistical significance**
Age (years)	51.48 ± 16.68	52.18 ± 13.10	NS
Sex (male/female)	46/35	21/19	NS
Body mass index	25.12 ± 4.06	25.35 ± 3.79	NS
Smoking	34 (42.5)	14 (35)	NS
Alcohol abuse	33 (40.74)	15 (37.5)	NS
Diabetes mellitus	16 (19.75)	9 (22.5)	NS
Chronic obstructive pulmonary disease	11 (13.58)	5 (12.5)	NS
Hypertension	29 (35.8)	14 (35)	NS
Traffic injury	44	–	–
Fall injury	19	–	–
Other injury	18	–	–

Data presented as *n* (%) prevalence or mean ± standard difference. NS, no statistically significant between-group difference (*p* > 0.05; χ^2^-test or Student's *t*-test).

### 3.5 Serum Nrf2 protein levels and their relationship with clinical severity and cytokines of sTBI

**Figure 4:**
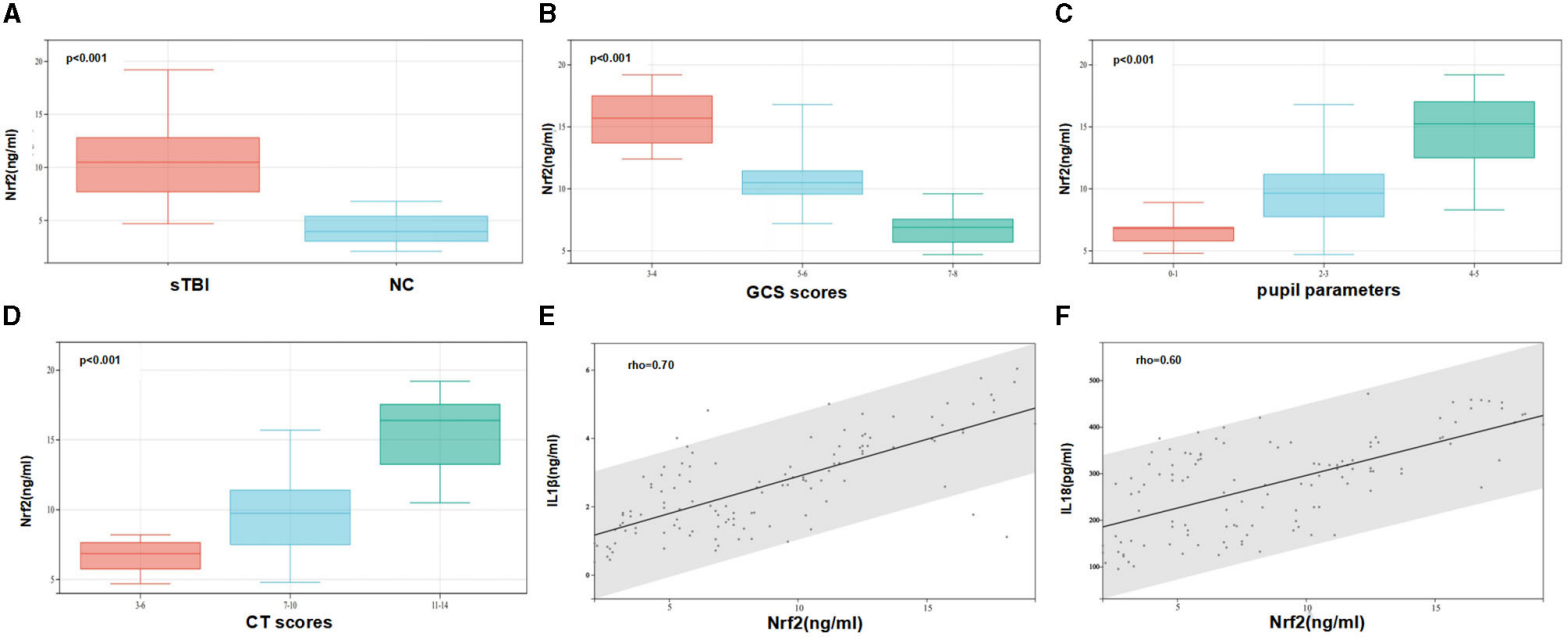
Serum Nrf2 protein levels and their relationship with clinical severity and cytokines of sTBI. **(A)** Comparison of serum levels of Nrf2 between normal control group and patients with sTBI. Patients with sTBI had significantly higher serum Nrf2 levels than healthy controls (*p* < 0.001). **(B)** The relationship between the level of Nrf2 and the GCS scores (*p* < 0.001). **(C)** The relationship between the level of Nrf2 and the level of pupil parameters (*p* < 0.001). **(D)** The relationship between the level of Nrf2 and the level of Helsinki CT Scores (*p* < 0.001). **(E)** The relationship between the level of Nrf2 and the level of IL1β. **(F)** The relationship between the level of Nrf2 and the level of IL18. Spearman's rank correlation test was performed to compare two variables. There is a strong negative association if the value of rho falls between −1 and −0.5. There is a strong positive association if the value of rho falls between 0.5 and 1.

Serum Nrf2 protein levels were measured using ELISA in 81 patients and 40 NCs. As shown in Figure 4A, Nrf2 protein levels were significantly higher in sTBI patients compared to NCs (*p* < 0.001).

To investigate the relationship between serum Nrf2 protein levels and disease severity of sTBI, the correlations between serum Nrf2 levels and GCS scores, pupil parameters, Helsinki CT scores were explored. The box plot showed that the levels of Nrf2 were reduced when the GCS scores was increased. There was negative relationship between the level of Nrf2 and the level of GCS (Figure 4B). In contrast, the levels of Nrf2 were increased when the levels of pupil parameters and Helsinki CT Scores were increased (Figures 4C, D). Spearman's rank correlation analysis showed that the levels of IL1β were increased when the level of Nrf2 was increased (Figure 4E). There was positive relationships between the level of Nrf2 and the level of IL1β. Similarly, the levels of IL18 were increased when the level of Nrf2 was increased (Figure 4F).

## 4 Discussion

In this paper, we utilized underutilized public data to identify potential biomarkers. The bioinformatic analysis laid the foundation for subsequent clinical research, and clinical study supported the results of the bioinformatic screen. We found that: ① Bioinformatic analysis suggested four hub genes, including NFE2L2, may play essential roles in pyroptosis following TBI; ② The Nrf2 levels in sTBI patients were significantly higher than those in healthy controls; ③ The serum Nrf2 levels were negatively associated with GCS scores and positively associated with pupil parameters, Helsinki CT scores, IL-1β, and IL-18. We speculate that serum Nrf2 may be used as a promising biochemical marker for evaluating severity of TBI.

Nrf2 is the transcription factor involved in regulating cellular oxidative stress response. Previous studies showed that Nrf2 is involved in numerous pathophysiological processes, including reducing oxidative stress, promoting mitochondrial biosynthesis, alleviating endoplasmic reticulum stress, and inhibiting ferroptosis. These pathological processes play important roles during secondary brain injury (16, 17). After brain trauma, Nrf2 translocates to the nucleus, activates expression of genes with neuroprotective function, and thereby leads to upregulation of expressions of various detoxification and anti-cell death factors, such as Bcl-2 and HSP70s. In some pre-clinical studies, Nrf2 knockdown significantly reduced the clearance of reactive oxygen species (ROS) both *in vivo* and *in vitro*, exacerbated secondary brain injury, thus provided theoretical support for Nrf2 to play its neuroprotective function (18–20). In our team's previous research, Nrf2 knockout [Nrf2 (–/–)] mice showed more severe neurological deficits, brain edema, and neuronal cell apoptosis compared to Nrf2 (+/+) mice after TBI. Nrf2 could partially ameliorate TBI induced secondary brain injury through the endoplasmic reticulum stress signaling pathway (19). We speculate that the increase in Nrf2 may represent a compensatory mechanism for the antistress ability of the organism. Severe stress, including trauma, activates effective self-protection, resulting in the increase in Nrf2.

Inflammatory cytokines are a group of small molecule proteins with extensive biological activity that regulate cell responses. As intercellular signaling molecules, inflammatory cytokines are produced and secreted by various cells and maintain body homeostasis. They are not only the needs of the body's stress response, but also the pathological basis of the occurrence and development of stressed tissue damage (21). Through the release of DAMPs, sometimes referred to as danger signals, brain damage can activate inflammasomes and innate immune responses (22). Endogenous DAMPs such as alarmins are released by nonapoptotic cells or immune cells (23). By binding to recipient cells, DAMPs trigger immune responses and release pro-inflammatory mediators. High mobility group box-1 (HMGB1) protein was found to translocate from the neuronal nucleus to the cytoplasm in the early hours after brain injury, then localized to microglia later (24). Activation of microglia and astrocytes, as well as infiltration of peripheral immune cells, further promoted central inflammatory responses (25). As important cytokines *in vivo*, IL1β, and IL18 play important roles in the progression and prognosis of many neurological diseases, including stroke and glioma (26, 27). Pre-clinical studies have investigated significant elevations of IL1β and IL18 after TBI in rodents (28).

Our study found a significant increase in serum Nrf2 levels in sTBI patients compared to healthy controls. The serum Nrf2 levels were negatively associated with GCS scores and positively associated with pupil parameters, Helsinki CT scores, IL-1β and IL-18. These results indicate that Nrf2, as an important protective factor in the body, is associated with the severity of the disease and affects the release of inflammatory cytokines. Therefore, our results provide an interesting future direction for investigating the underlying mechanisms.

Despite robust findings, there are still some limitations. One limitation of this study consists of the retrospective single-center design. The large-scale, multicenter, prospective studies are needed. Besides this, some potential confounding factors, such as emergency surgery, may be related to the expression of Nrf2 and the secretion of cytokines. Our study did not evaluate these variables, so it is recommended to investigate these factors in the future studies. There was no long-term follow-up to assess clinical outcomes, so no multivariate prognostic analysis was conducted. Our findings should be understood with caution. Follow-up research is needed to observe long-term outcomes.

## 5 Conclusions

In conclusion, we used various bioinformatic analyses to screen four hub genes, including NFE2L2, may play important role in the pathology of TBI. The subsequent clinical research indicated the increase in serum Nrf2 levels was closely related to the severity of trauma and cytokines. To some extent, these results provide more detailed and realistic evidence for the role of Nrf2 in TBI. We speculate that serum Nrf2 may serve as a promising biochemical marker for the assessment of TBI in clinical practice.

## Data availability statement

The original contributions presented in the study are included in the article/Supplementary material, further inquiries can be directed to the corresponding author.

## Ethics statement

The studies involving humans were approved by Harrison International Peace Hospital review board. The studies were conducted in accordance with the local legislation and institutional requirements. Written informed consent was not required to participate in this study in accordance with the local legislation and institutional requirements.

## Author contributions

GZ: Conceptualization, Data curation, Funding acquisition, Methodology, Writing—original draft, Writing—review & editing. JZ: Data curation, Visualization, Writing—original draft. JL: Data curation, Visualization, Writing—original draft. GS: Conceptualization, Funding acquisition, Supervision, Writing—original draft, Writing—review & editing.
