# Supplementary material for: Nrf2 functions as a pyroptosis-related mediator in traumatic brain injury and is correlated with cytokines and disease severity: a bioinformatics analysis and retrospective clinical study
Source: Front Neurol. 2024 Feb 9;15:1341342. doi: 10.3389/fneur.2024.1341342 (PMC10884226; doi:10.3389/fneur.2024.1341342)
Supplement: Supplementary file 1 [file Table_1.doc]

Supplemental Table 1. The pupil evaluation

| Category | Findings | Score |
| --- | --- | --- |
| Pupil reactivity | Brisk | 0 |
|  | Sluggish | 1 |
|  | Fixed | 3 |
| Pupil size | Normal | 0 |
|  | Anisocoric | 1 |
|  | Bilateral dilated | 2 |
