# Supplementary material for: Nrf2 functions as a pyroptosis-related mediator in traumatic brain injury and is correlated with cytokines and disease severity: a bioinformatics analysis and retrospective clinical study
Source: Front Neurol. 2024 Feb 9;15:1341342. doi: 10.3389/fneur.2024.1341342 (PMC10884226; doi:10.3389/fneur.2024.1341342)
Supplement: Supplementary file 2 [file Table_2.docx]

**Supplemental Table 2.**

**Pyroptosis-related genes with relevance score≥2**

| GSDMD | AIM2 | IL18 | MIR223 | HOTTIP | GBP1 |
| --- | --- | --- | --- | --- | --- |
| GSDME | CASP8 | H19 | APIP | STAT3 | MIR155 |
| NLRP3 | IL1B | GZMA | MIR7-3HG | LINC02605 | CERNA3 |
| CASP1 | GAS5 | XIST | NLRC4 | SNHG7 | CASP7 |
| CASP4 | GZMB | DPP9 | DPP8 | DDX3X | ZFAS1 |
| GSDMB | KCNQ1OT1 | SOD2-OT1 | TMX2-CTNND1 | MEFV | BDNF-AS |
| GSDMC | MEG3 | ZBP1 | HMGB1 | TRIM24 | MAP3K20 |
| NLRP1 | CASP5 | TRIM21 | IFI27 | AGER | SIRT1 |
| CASP3 | PYCARD | CASP6 | SCARNA5 | NAIP | PVT1 |
| GSDMA | CARD8 | ADAMTS9-AS2 | MALAT1 | MAPK14 | DHX9 |
| ELAVL1 | MIR155HG | MAPK11 | HIF1A-AS1 | MIR30C1 | TNF |
| TXNIP | MIR216A | PGR-AS1 | NEK7 | TREM2 | VEGFA |
| MIR125A | NLRP6 | SNHG12 | SNORD118 | CTSG | TUG1 |
| NLRP9 | TP53 | NEAT1 | FOXO3 | NFKB1 | OIP5-AS1 |
| KLF3-AS1 | H2AX | TP63 | LINC00339 | LINC01871 | MIR34C |
| MIAT | H2AC20 | SMAD5-AS1 | MIR21 | NFE2L2 | HOTAIRM1 |
| LINC01672 | ELANE |  |  |  |  |
