# Supplementary material for: Nrf2 functions as a pyroptosis-related mediator in traumatic brain injury and is correlated with cytokines and disease severity: a bioinformatics analysis and retrospective clinical study
Source: Front Neurol. 2024 Feb 9;15:1341342. doi: 10.3389/fneur.2024.1341342 (PMC10884226; doi:10.3389/fneur.2024.1341342)
Supplement: Supplementary file 3 [file Table_3.docx]

**Supplemental Table 3**.

**Differentially expressed pyroptosis-related genes(DEPGs)**

| NLRP3 | NLRC4 | SNHG12 | MIR155HG |
| --- | --- | --- | --- |
| GAS5 | NAIP | FOXO3 | NFE2L2 |
| CASP6 | MAPK14 | TXNIP |  |
