# Supplementary material for: Nrf2 functions as a pyroptosis-related mediator in traumatic brain injury and is correlated with cytokines and disease severity: a bioinformatics analysis and retrospective clinical study
Source: Front Neurol. 2024 Feb 9;15:1341342. doi: 10.3389/fneur.2024.1341342 (PMC10884226; doi:10.3389/fneur.2024.1341342)
Supplement: Supplementary file 4 [file Table_4.doc]

**Supplemental Table 4.**

**Functional and pathway enrichment analysis of DEPGs**

| Category | Term | Count | P-value |
| --- | --- | --- | --- |
| Biological processes | pyroptosis | 4 | 0.00000058 |
| Biological processes | inflammatory response | 6 | 0.0000013 |
| Biological processes | apoptotic process | 6 | 0.0000071 |
| Biological processes | pattern recognition receptor signaling pathway | 3 | 0.000025 |
| Cellular components | IPAF inflammasome complex | 2 | 0.0026 |
| Cellular components | cytoplasm | 8 | 0.0028 |
| Cellular components | inflammasome complex | 2 | 0.0048 |
| Cellular components | cytosol | 7 | 0.016 |
| Molecular functions | sequence-specific DNA binding | 3 | 0.0086 |
| Molecular functions | transcription cofactor binding | 2 | 0.015 |
| Molecular functions | ATP binding | 4 | 0.022 |
| Molecular functions | transcription regulatory region sequence-specific DNA binding | 2 | 0.096 |
| KEGG pathway | NOD-like receptor signaling pathway | 5 | 0.0000071 |
| KEGG pathway | Shigellosis | 5 | 0.000022 |
| KEGG pathway | Lipid and atherosclerosis | 4 | 0.0005 |
| KEGG pathway | Salmonella infection | 4 | 0.00077 |
| KEGG pathway | Yersinia infection | 3 | 0.005 |
| KEGG pathway | Pathogenic Escherichia coli infection | 3 | 0.01 |
| KEGG pathway | Chemical carcinogenesis - reactive oxygen species | 3 | 0.013 |
| KEGG pathway | Legionellosis | 2 | 0.045 |
