# Supplementary material for: Nrf2 functions as a pyroptosis-related mediator in traumatic brain injury and is correlated with cytokines and disease severity: a bioinformatics analysis and retrospective clinical study
Source: Front Neurol. 2024 Feb 9;15:1341342. doi: 10.3389/fneur.2024.1341342 (PMC10884226; doi:10.3389/fneur.2024.1341342)
Supplement: Supplementary file 5 [file Table_5.doc]

Supplemental Table 5.CytoHubba hub genes screened.

| BottleNeck | MCC | EcCentricity | EPC |
| --- | --- | --- | --- |
| **NFE2L2** | **NLRP3** | **TXNIP** | **NLRP3** |
| **TXNIP** | **TXNIP** | **NFE2L2** | **TXNIP** |
| **NLRP3** | **NFE2L2** | **NLRP3** | **NFE2L2** |
| **NLRC4** | **NLRC4** | **NLRC4** | **NLRC4** |
| CASP6 | NAIP | CASP6 | FOXO3 |
| NAIP | FOXO3 | NAIP | NAIP |
| FOXO3 | CASP6 | FOXO3 | CASP6 |
| MAPK14 | MAPK14 | MAPK14 | MAPK14 |

Bold gene symbols were the hub genes in top 4 by four ranked methods respectively in cytoHubba. EPC: Edge Percolated Component; MCC:Maximal Clique Centrality.
