# Supplementary material for: Nrf2 functions as a pyroptosis-related mediator in traumatic brain injury and is correlated with cytokines and disease severity: a bioinformatics analysis and retrospective clinical study
Source: Front Neurol. 2024 Feb 9;15:1341342. doi: 10.3389/fneur.2024.1341342 (PMC10884226; doi:10.3389/fneur.2024.1341342)
Supplement: Supplementary file 6 [file Data_Sheet_1.doc]

**Supplementary Appendix 1**

**The search strings for pyroptosis in the GeneCard**

[aliases] ( pyroptosis ) OR [compounds] ( pyroptosis ) OR [disorders] ( pyroptosis ) OR [inferreddisorders] ( pyroptosis ) OR [domains] ( pyroptosis ) OR [expression] ( pyroptosis ) OR [functions] ( pyroptosis ) OR [genomics] ( pyroptosis ) OR [localization] ( pyroptosis ) OR [orthologs] ( pyroptosis ) OR [paralogs] ( pyroptosis ) OR [pathways] ( pyroptosis ) OR [phenotypes] ( pyroptosis ) OR [proteins] ( pyroptosis ) OR [publications] ( pyroptosis ) OR [summaries] ( pyroptosis ) OR [variants] ( pyroptosis ) OR [transcripts] ( pyroptosis )
